# Association between brain cancer immunogenetic profile and in silico immunogenicities of 11 viruses

**DOI:** 10.1038/s41598-023-48843-6

**Published:** 2023-12-06

**Authors:** Apostolos P. Georgopoulos, Lisa M. James

**Affiliations:** 1 The HLA Research Group, Brain Sciences Center, Department of Veterans Affairs Health Care System, Minneapolis VAMC, One Veterans Drive, Minneapolis, MN 55417 USA; 2 Department of Neuroscience, University of Minnesota Medical School, Minneapolis, MN USA; 3 Department of Psychiatry, University of Minnesota Medical School, Minneapolis, MN USA; 4 Department of Neurology, University of Minnesota Medical School, Minneapolis, MN USA

**Keywords:** Cancer, Computational biology and bioinformatics, Immunology, Medical research, Neurology, Oncology, Pathogenesis

## Abstract

Several viruses including human herpes viruses (HHVs), human polyomavirus JCV, and human papilloma virus (HPV) have been implicated in brain cancer, albeit inconsistently. Since human leukocyte antigen (HLA) is centrally involved in the human immune response to viruses and has been implicated in brain cancer, we evaluated in silico the immunogenicity between 69 Class I HLA alleles with epitopes of proteins of 9 HHVs, JCV, and HPV with respect to a population-based HLA-brain cancer profile. We found that immunogenicity varied widely across HLA alleles with HLA-C alleles exhibiting the highest immunogenicity, and that immunogenicity scores were negatively associated with the population-based HLA-brain cancer profile, particularly for JCV, HHV6A, HHV5, HHV3, HHV8, and HHV7. Consistent with the role of HLA in foreign antigen elimination, the findings suggest that viruses with proteins of high HLA immunogenicity are eliminated more effectively and, consequently, less likely to cause brain cancer; conversely, the absence of highly immunogenic HLA may allow the viral antigens to persist, contributing to cancer.

## Introduction

Investigation into the role of viral infections on brain cancer have supported an influence of several common viruses on brain cancer risk and prognosis. For example, human cytomegalic virus (HHV5), human polyoma JC virus (JCV) and human papilloma virus (HPV) have been widely implicated in brain cancers and have been detected in brain tumors, albeit inconsistently^1–8^. Still other viruses, particularly other human herpes viruses (HHV) such as HHV1 and HHV2 (herpes simplex viruses), HHV4 (Epstein–Barr virus), and HHV6 (Roseolovirus) have been cited as possibly linked to brain cancers although research is relatively sparse and inconsistent^1, 2, 4, 8, 9^. Some of the inconsistencies with regard to the associations of viruses with brain cancer might be partially attributable to population^10, 11^ and individual^12^ variation in Class I human leukocyte antigen (HLA) genes, an essential component of the human immune response to viruses and other foreign antigens.

Class I HLA genes code for cell-surface proteins located on nucleated cells that bind and export small peptides derived from proteolytically degraded cytosolic viruses and other foreign antigens to the cell surface for presentation to CD8+ cytotoxic T cells, thereby signaling cell destruction. The success of this process rests on both the binding affinity of an HLA molecule with a foreign antigen epitope and immunogenicity, or the ability to mount an immune response. HLA is the most highly polymorphic region of the human genome with nearly all of the variability located in the binding groove^12^; consequently, subtle differences in the amino acid residues of the binding groove influence antigen elimination^13^. Exposure to viral or other foreign antigens in the absence of high affinity HLA-antigen binding or insufficient immunogenicity may result in antigen persistence and downstream deleterious health effects^14, 15^. Indeed, HLA has been implicated in a wide variety of conditions including cancers^16–31^ and, specifically, has been implicated in brain cancer risk and protection^32–39^.

Taken together, separate lines of research have linked several viruses and HLA to brain cancer. We have suggested that HLA moderates the influence of exposure to viruses on long-term health effects including cancer such that HLA-facilitated antigen elimination promotes health and, conversely, the inability to eliminate foreign antigens due to weak binding affinity and/or immunogenicity, contributes to cancer risk^27–31^. Here, we evaluated HLA-virus immunogenicity with respect to a population-based HLA-brain cancer profile. Specifically, we computed an HLA profile for brain cancer characterized by the covariance between the population frequency of HLA alleles and the population prevalence of brain cancer in 14 Continental Western European countries. Next, we used an in silico approach to evaluate the binding and immunogenicity of viral antigens linked to brain cancer with common HLA Class I alleles and to assess the association between those HLA-antigen epitope immunogenicity scores and the HLA-brain cancer profile.

## Results

### Brain Cancer-HLA protection/susceptibility covariance (BC-HLA PScov) scores

The BC-HLA PScov scores are given in Table 1. We rejected the null hypothesis that this set of PScov scores could be due to chance, since no cases were found where the ranks following a random pairing of BC-prevalence with allele frequency (out of 1,000,000 runs) matched the ranked observed BC-HLA PScov profile, thus rejecting the null hypothesis that the observed profile could be accounted for by chance (P < 1 × 10^–6^).

**Table 1:** Brain cancer PScov × 1000 scores for HLA Class I alleles.

	Allele	BC PScov
1	A*01:01	− 0.2952
2	A*02:01	0.5382
3	A*02:05	− 0.1039
4	A*03:01	1.1290
5	A*11:01	− 0.0594
6	A*23:01	− 0.1914
7	A*24:02	0.1594
8	A*25:01	− 0.1075
9	A*26:01	− 0.1197
10	A*29:01	0.0682
11	A*29:02	− 0.2047
12	A*30:01	− 0.1013
13	A*30:02	− 0.0785
14	A*31:01	0.2128
15	A*32:01	− 0.0159
16	A*33:01	− 0.1004
17	A*33:03	0.0166
18	A*36:01	− 0.0162
19	A*68:01	0.0510
20	A*68:02	− 0.2907
21	B*07:02	0.3402
22	B*08:01	0.0555
23	B*13:02	− 0.0664
24	B*14:01	− 0.0576
25	B*14:02	− 0.2176
26	B*15:01	0.3654
27	B*15:17	− 0.0292
28	B*15:18	0.0206
29	B*18:01	0.1684
30	B*27:02	0.0699
31	B*27:05	0.5210
32	B*35:01	0.2589
33	B*35:02	− 0.0911
34	B*35:03	− 0.0715
35	B*35:08	0.0366
36	B*37:01	0.0889
37	B*38:01	− 0.0487
38	B*39:01	0.0261
39	B*39:06	0.0478
40	B*40:01	0.5888
41	B*40:02	0.1338
42	B*41:01	0.0567
43	B*41:02	0.0026
44	B*44:02	− 0.1702
45	B*44:03	− 0.3708
46	B*44:05	0.0159
47	B*45:01	− 0.0031
48	B*47:01	0.0071
49	B*49:01	− 0.1197
50	B*50:01	− 0.1328
51	B*51:01	0.3583
52	B*52:01	0.1448
53	B*55:01	− 0.0422
54	B*56:01	0.0712
55	B*57:01	− 0.0430
56	B*58:01	0.0435
57	C*01:02	0.2524
58	C*03:03	0.5642
59	C*04:01	− 0.1252
60	C*05:01	− 0.1708
61	C*06:02	− 0.3901
62	C*07:01	0.2317
63	C*07:02	0.3645
64	C*07:04	− 0.0421
65	C*12:02	0.0610
66	C*12:03	− 0.2835
67	C*14:02	− 0.0862
68	C*15:02	− 0.1222
69	C*16:01	− 0.3437

### Immunogenicity of virus proteins for HLA Class I alleles

**Figure 1:**
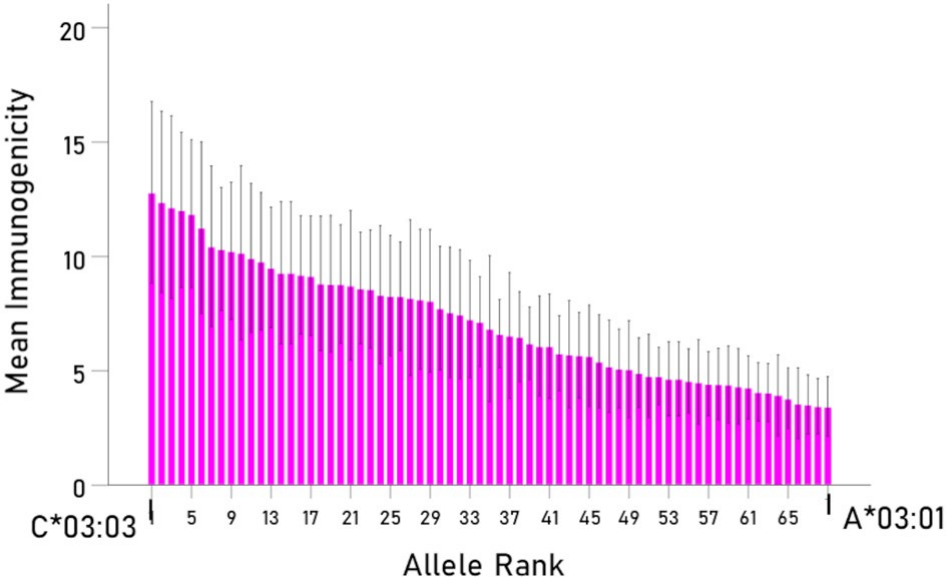
Mean immunogenicity scores *I* (± 95% CI) of the 69 HLA alleles used, ranked from high to low. The number in the abscissa correspond to the ranks in Table 3 (column 1). See text for details.

**Figure 2:**
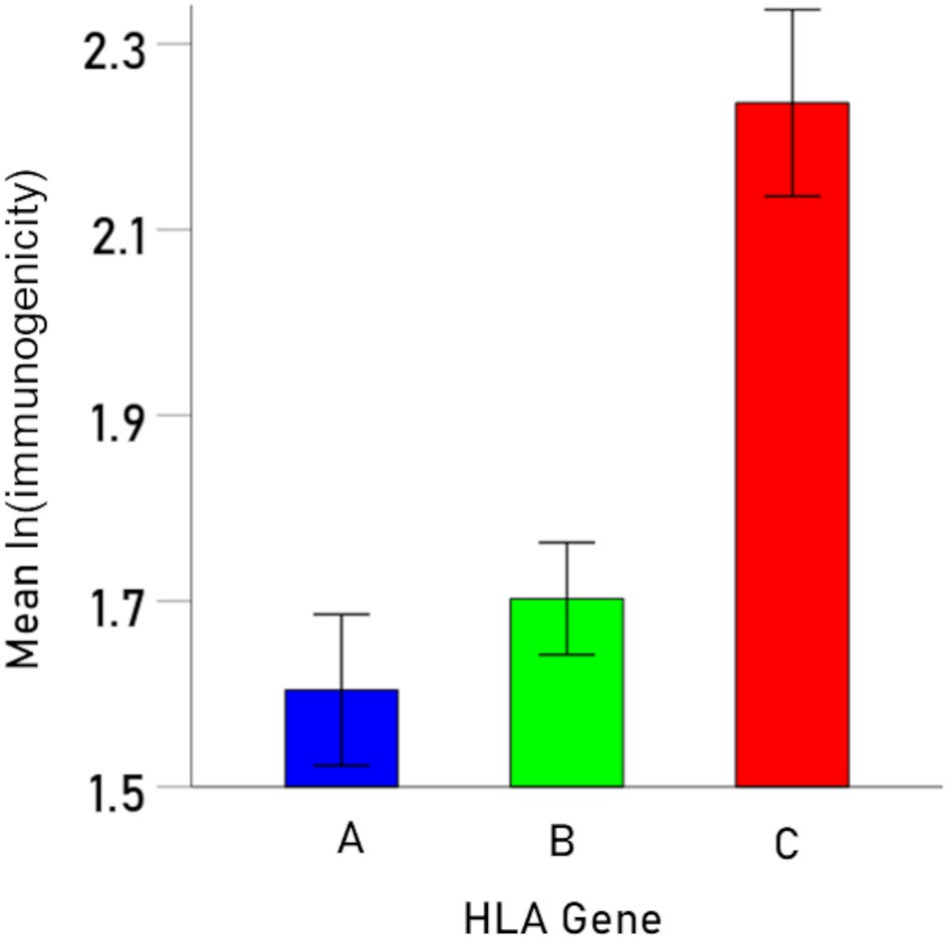
Mean (± 95% CI) log-transformed immunogenicity score ($I^{\prime}$) for each one of the 3 HLA genes. See text for detail.

Immunogenicity scores varied appreciably among HLA alleles (Fig. 1 and Table 2). Allele C*03:03 had the highest *I* score (12.81) and A*03:01 had the lowest score (3.45), a 3.7 × differential. An analysis of variance (ANOVA) was used to evaluate the effect of HLA Gene on the log-transformed immunogenicity score $I^{\prime}$. We found that Gene had a highly statistically significant (F_[2,754]_ = 51.9, P < 0.001). It can be seen in Fig. 2 that HLA allele C had the highest immunogenicity effect (P < 0.001 for both comparisons against genes A and B; two-sided t-test ANOVA), whereas gene B had a higher effect than gene A which, however, did not reach statistical significance (P = 0.057, two-sided t-test, ANOVA). Finally, given that an individual carries 6 classical HLA Class I alleles (2 for A, B, and C genes), the combination of alleles with highest average immunogenicity are shown in bold in Table 2.

**Table 2:** Average immunogenicity score *I* for the 69 HLA Class I alleles, sorted by gene and ranked from high to low (N = 11 viruses).

Gene A		Gene B		Gene C	
Allele	*I*	Allele	*I*	Allele	*I*
A*68:02	**10.45**	B*56:01	**9.79**	C*03:03	**12.81**
A*02:05	**10.34**	B*55:01	**9.52**	C*12:03	**12.39**
A*32:01	8.8	B*14:01	9.30	C*12:02	12.16
A*25:01	8.2	B*14:02	9.30	C*01:02	12.04
A*30:01	7.74	B*35:02	9.16	C*16:01	11.87
A*02:01	7.26	B*52:01	8.81	C*15:02	11.27
A*26:01	6.85	B*13:02	8.62	C*14:02	10.25
A*23:01	6.21	B*35:03	8.58	C*07:04	10.17
A*24:02	5.77	B*15:18	8.34	C*04:01	9.94
A*33:01	5.41	B*35:08	8.29	C*05:01	9.21
A*31:01	5.20	B*35:01	8.28	C*07:02	8.83
A*33:03	5.08	B*39:01	8.07	C*06:02	8.74
A*68:01	4.78	B*38:01	7.56	C*07:01	8.13
A*29:01	4.66	B*39:06	7.47		
A*29:02	4.66	B*07:02	7.16		
A*36:01	4.41	B*51:01	6.63		
A*01:01	3.93	B*47:01	6.55		
A*30:02	3.80	B*15:17	6.50		
A*11:01	3.54	B*18:01	6.09		
A*03:01	3.45	B*37:01	6.09		
		B*58:01	5.73		
		B*15:01	5.69		
		B*57:01	5.66		
		B*50:01	5.10		
		B*49:01	4.92		
		B*08:01	4.78		
		B*41:01	4.56		
		B*45:01	4.51		
		B*44:03	4.45		
		B*40:01	4.43		
		B*40:02	4.33		
		B*44:02	4.28		
		B*41:02	4.08		
		B*44:05	4.06		
		B*27:05	3.58		
		B*27:02	3.47		

The combination of 6 alleles with the highest immunogenicity scores are shown in bold.

### Association between BC-HLA PScov score and virus proteins immunogenicity

A main objective of this study was to assess in silico the possible association between BC-HLA PScov scores and the HLA-immunogenicity of the proteins of various viruses that have been implicated in brain cancer. Our rationale was that viruses with proteins of high HLA immunogenicity would be eliminated more effectively from the body and the brain, and hence less likely to cause cancer: this hypothesis would predict that BC-HLA PScov scores would be negatively correlated with HLA virus immunogenicity. Indeed, the immunogenicity scores ${I}^{\prime}$ of virus proteins were, overall, negatively and highly significantly associated with the BC-HLA PScov scores (r = − 0.126, P < 0.001, N = 11 proteins × 69 alleles = 759).

**Figure 3:**
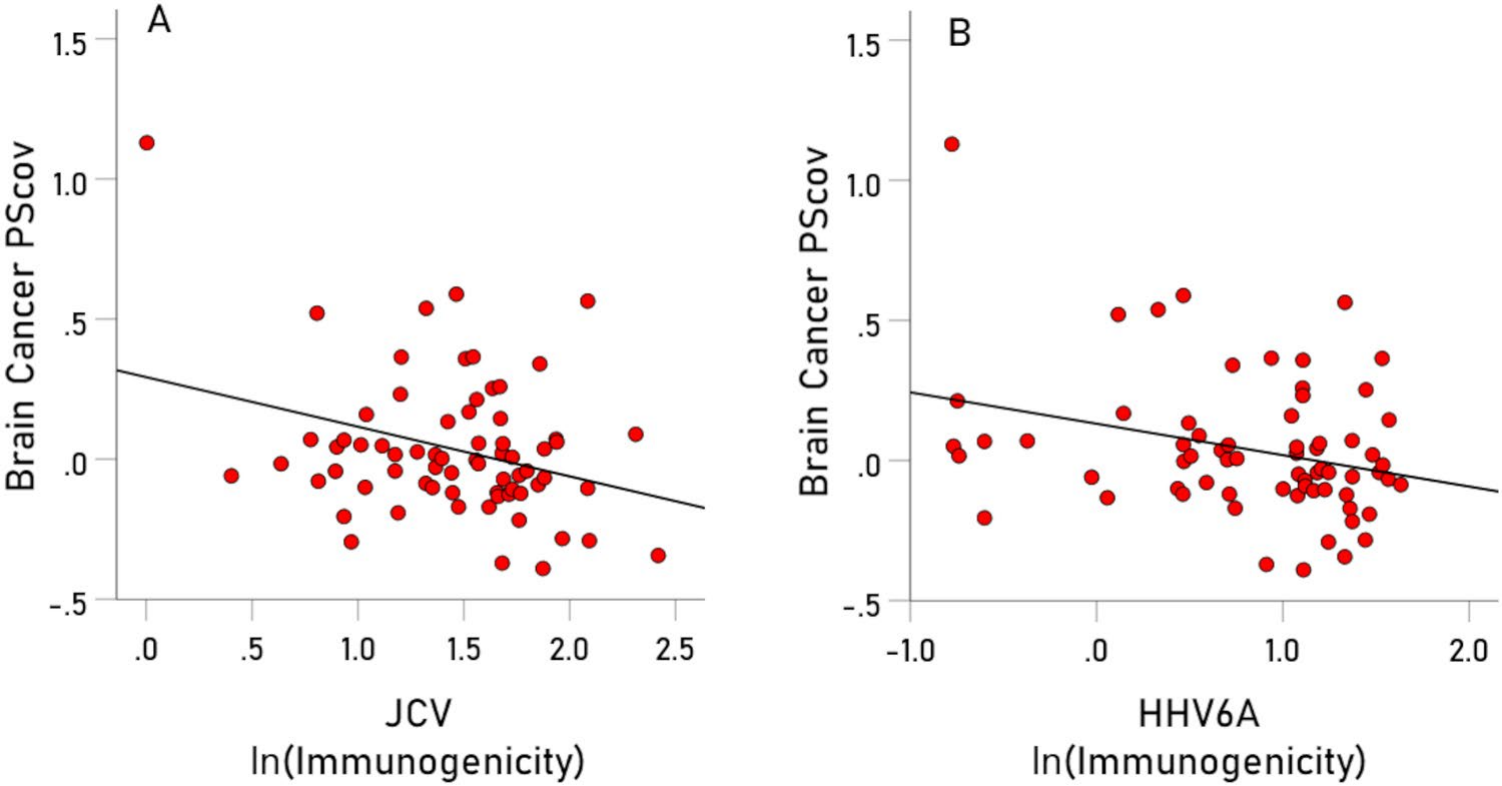
(**A**) BC-HLAcov scores are plotted against $I^{\prime}$ for JCV virus. (**B**) Same for HHV6A virus. See Table 3 for statistics.

**Figure 4:**
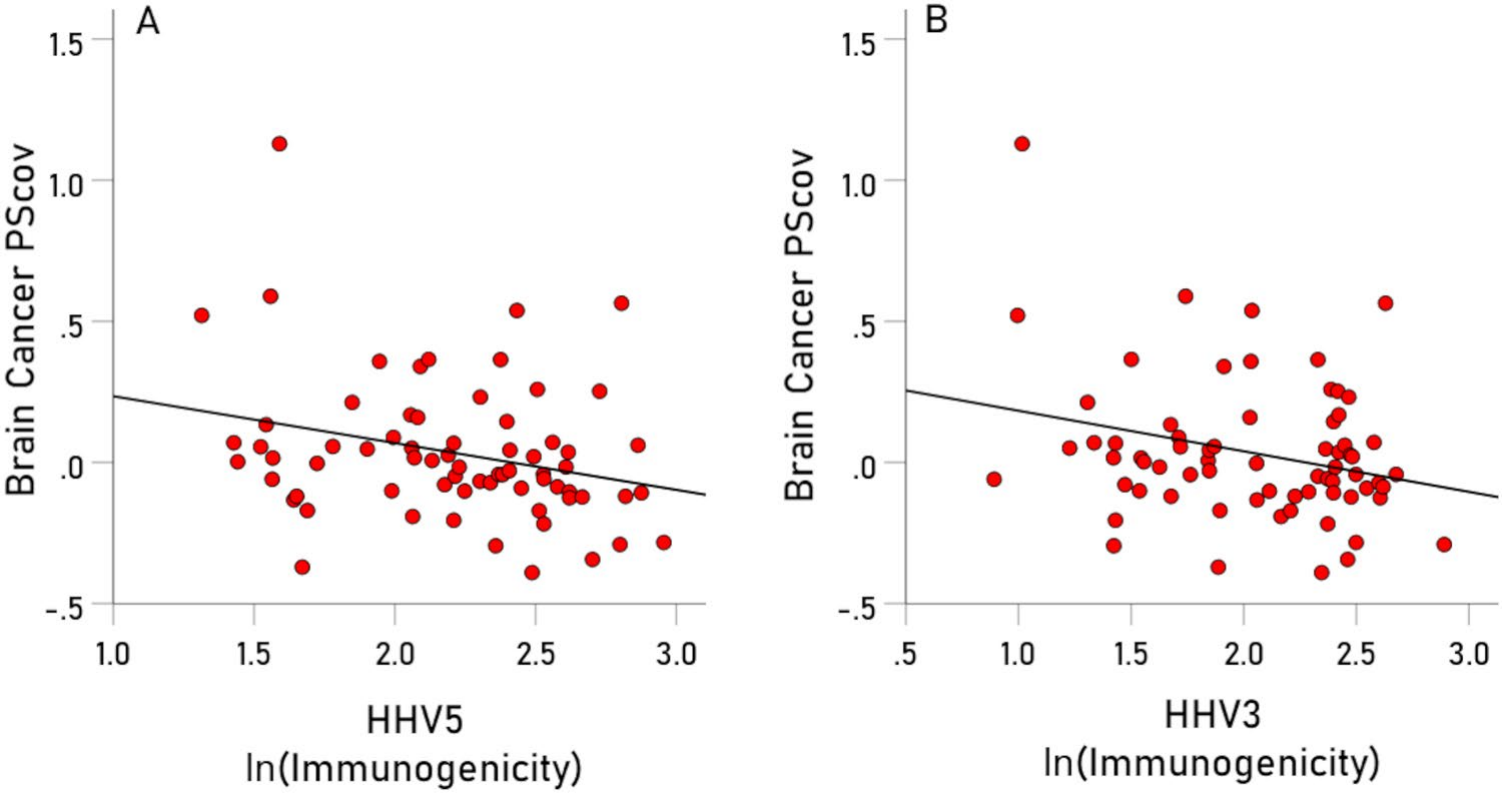
(**A**) BC-HLAcov scores are plotted against $I^{\prime}$ for HHV5 virus. (**B**) same for HHV3 virus. See Table 3 for statistics.

**Figure 5:**
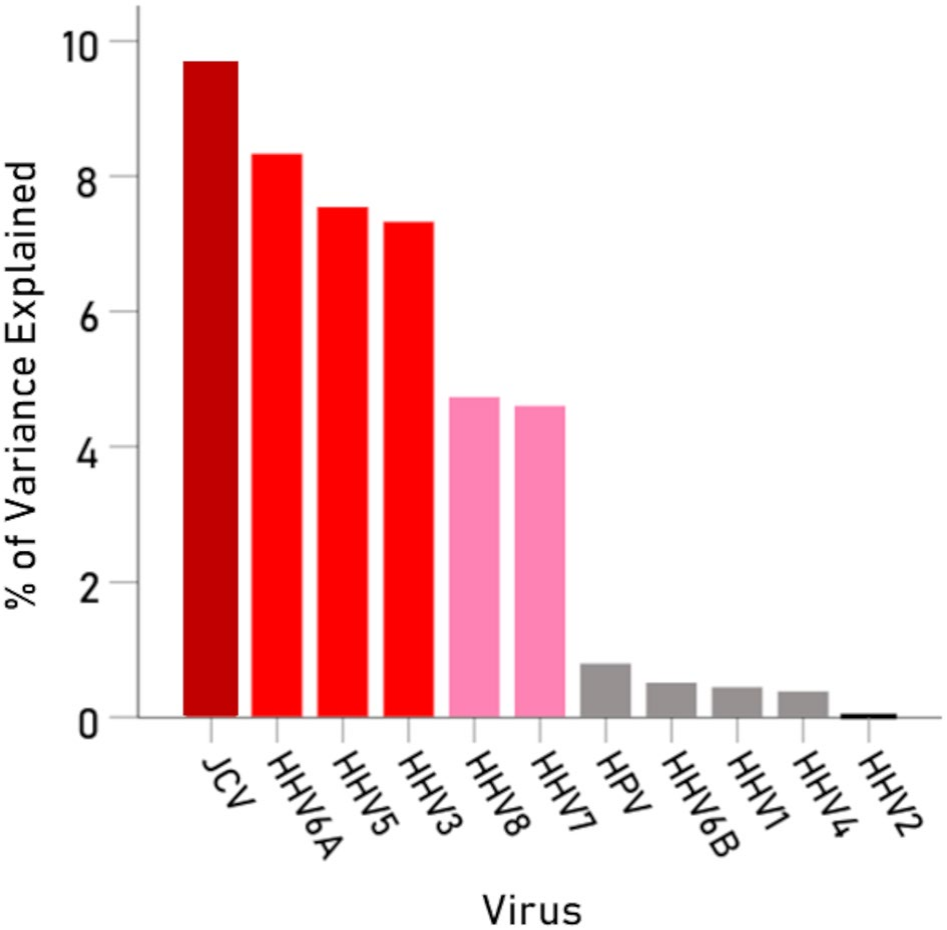
Percent of variance of BC PScov scores explained by immunogenicities of the 11 viruses studied. Colors reflect gradation in PVE. (Data from Table 3).

**Figure 6:**
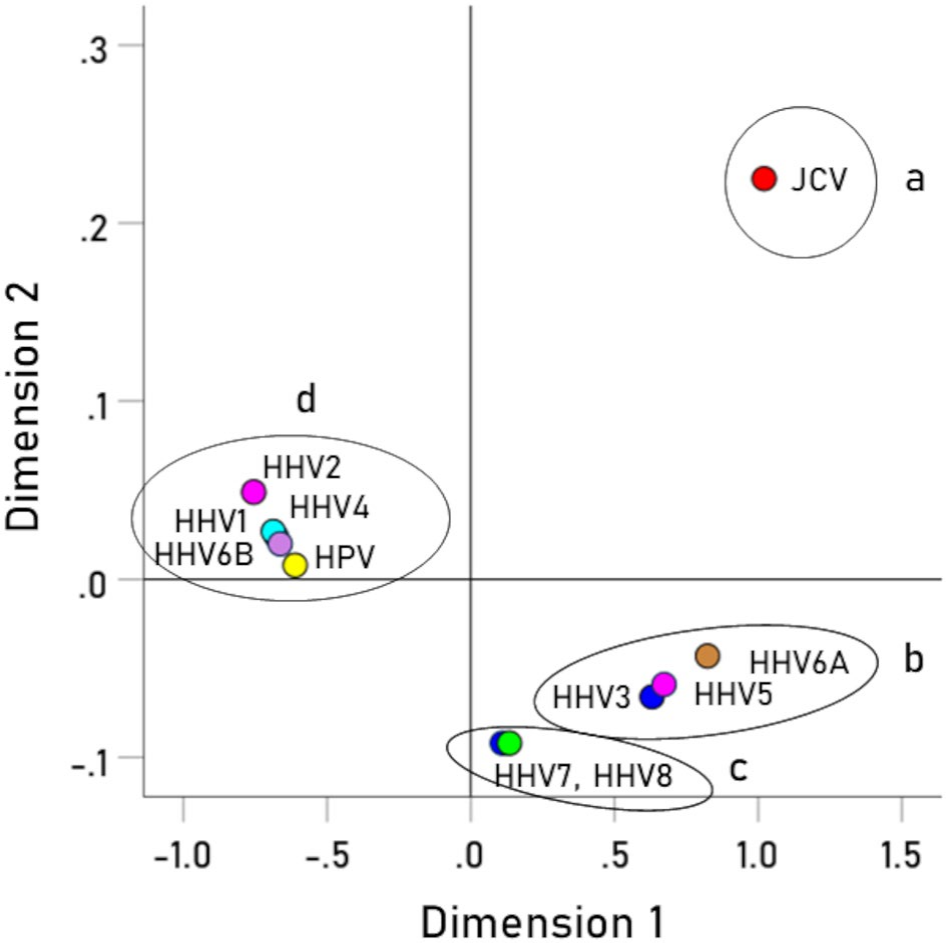
Multidimensional scaling plot of percent variance explained by the 11 viruses studied. See text for details.

**Figure 7:**
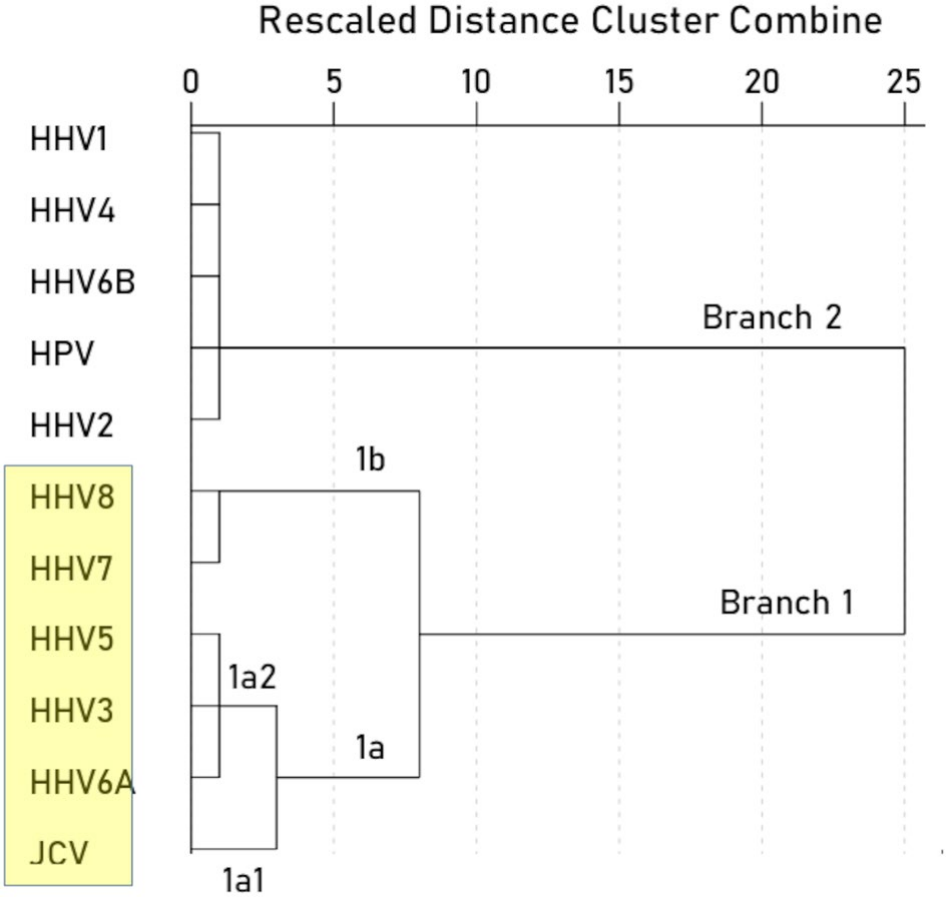
Dendrogram derived by hierarchical tree clustering of percent variance explained by the 11 viruses studied. See text for details.

**Figure 8:**
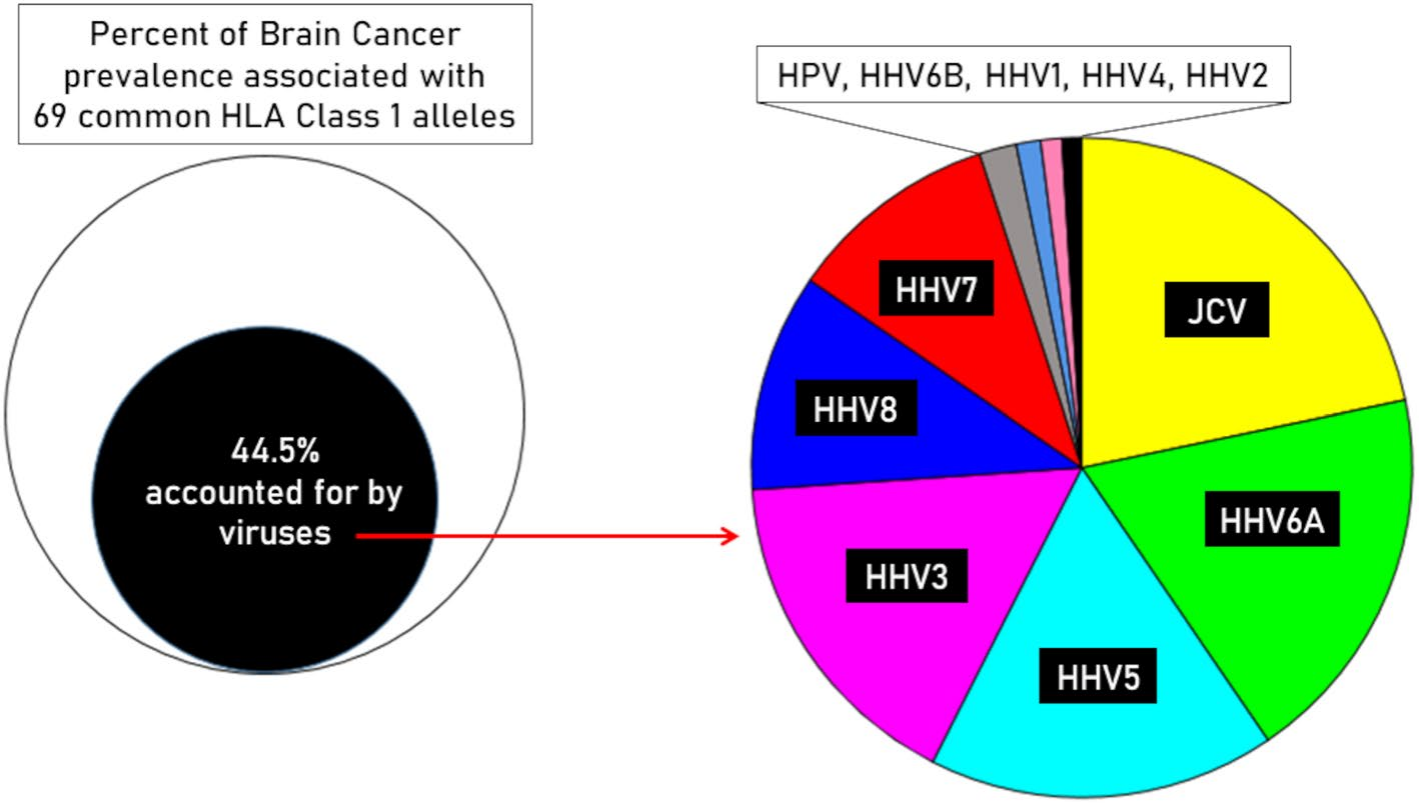
Schematic diagrams to illustrate the contributions of the 11 viruses studied to the BC-HLA association. The filled black circle in the left panel denotes the percentage of the BC-HLA variance explained by the sum of contributions by immunogenicities of individual viruses (column 3 in Table 3). The area of the color-coded viral sectors in the right panel is proportional to individual viral contributions shown in column 3 of Table 3. The sectors of HHV4 and HHV2 (in black) were almost totally overlapping.

With respect to specific viruses, the key measure was the percent of the BC-HLA PScov scores explained by the virus immunogenicity (Percent Variance Explained, PVE). The results are shown in Table 3, ranked from high to low PVE, and illustrated in Figs. 3, 4, 5, 6, 7, 8. The following can be seen. Ten of the 11 virus immunogenicities (all but HPV) were negatively associated with the corresponding (per HLA allele) BC-HLA PScov scores. JCV had the highest PVE (column 3 in Table 3, Fig. 3A), followed by HHV6A (Fig. 3B), HHV5 (Fig. 4A), HHV3 (Fig. 4B), HHV8, and HHV7, whereas HPV, HHV6B, HHV4, and HHV2 had negligible PVEs (all < 1%). These groupings of BC-HLA and virus immunogenicity associations are illustrated in Fig. 5 which plots the PVE (Eq. 4) for each virus. A formal assessment of the PVE using MDS differentiated the groupings more clearly. It can be seen in Fig. 6 that (a) JCV is alone in the upper right quadrant, (b) HHV6A, HH5 and HHV3 are in the lower right quadrant, (c) HHV7 and HHV8 are in a separate cluster also in the lower right quadrant, and (d) all the remaining viruses (HHV1, HHV2, HHV4, HHV6B, and HPV) are tightly clustered together in the upper left quadrant. This segregation of PVEs is further exemplified in the dendrogram of Fig. 7, as follows. There are two main branches: (i) branch 1 comprises the 6 viruses with non-negligible association to BC-HLA scores, corresponding to the points on the positive (right) side of the MDS plot of Fig. 6 (clusters a, b, c); and (ii) branch 2 comprises the 5 viruses with negligible contributions (PVE) to BC-HLA scores (corresponding to cluster d in Fig. 6). Furthermore, branch 1 gives rise to two smaller branches (1a and 1b). Branch 1a comprises two branches (1a1 and 1a2), of which branch 1a1 contains JCV alone (corresponding to cluster a in Fig. 6) and branch 1a2 contains HHV3, HHV5 and HHV6A (corresponding to cluster b in Fig. 6); branch 1b comprises HHV7 and HHV8 (cluster c in Fig. 6).

**Table 3:** Pearson correlations (and associated statistics) between BC PScov and the log-transformed immunogenicity of the 11 virus proteins studied.

Virus proteins from Table 6					
Rank	Virus	Correlation	% Variance explained (PVE)	% Contribution	P-value
**1**	**JCV**	**− 0.311**	**9.67**	**21.72**	**0.005**
**2**	**HHV6A**	**− 0.289**	**8.35**	**18.76**	**0.009**
**3**	**HHV5 gB**	**− 0.275**	**7.56**	**16.98**	**0.011**
**4**	**HHV3**	**− 0.271**	**7.34**	**16.49**	**0.012**
**5**	**HHV8**	**− 0.218**	**4.75**	**10.67**	**0.036**
**6**	**HHV7**	**− 0.215**	**4.62**	**10.38**	**0.038**
7	HPV	− 0.090	0.81	1.82	0.232
8	HHV6B	− 0.073	0.53	1.19	0.277
9	HHV1	− 0.068	0.46	1.03	0.288
10	HHV4	− 0.063	0.40	0.9	0.304
11	HHV2	0.017	0.03	0.07	0.446
			Total 44.52%	100%	

Additional HHV5/CMV proteins from Table 7					
	Protein	Correlation (P-value)	% Variance explained (PVE)		
1	pp65 (AD169)	− 0.180 (P = 0.069)	3.24		
2	pp65 (Merlin)	− 0.188 (P = 0.061)	3.53		
3	pp65 (Towne)	− 0.189 (P = 0.060)	3.57		
4	gM HCMVA	− 0.168 (P = 0.086)	2.82		
5	gN HCMVA	− 0.085 (P = 0.261)	0.72		

P-values are 1-sided. HHV, human herpes virus; JCV, human polyomavirus; HPV human papillomavirus.

Significant values are in bold.

The total, combined PVE of the 11 viruses was 44.52%, which means that of the BC-HLA PScov variance, 44.52% was accounted for by the immunogenicities of the 11 virus proteins studied (Fig. 8, left panel). The relative contributions to this total by each virus are given in column 4 of Table 3 and illustrated in the pie graph on the right panel in Fig. 8. Specifically, PVEs of JCV, HHV6A, HHV5, HHV3, HHV8, and HHV7 contributed 95% of PVE, each virus contributing > 10%. In contrast, the remainder viruses (HPV, HHV6B, HHV4, HHV2) contributed only 5%, each virus contributing < 2%. Finally, this sharp separation of the two groups of viruses was also reflected in the significance levels of the correlation coefficients. (One-sided P-values were used because we were testing explicitly only a negative association between virus immunogenicity and BC-HLA covariance, and no correction for multiple comparisons was applied because these were planned comparisons and not the results of a dredging search for significant correlations.)

### Additional HHV5 proteins

The immunogenicity scores of all 5 additional HHV5 proteins were negatively correlated with the BC-HLA PScov scores but at strengths smaller than that of HHV5 gB (Table 3, lower panel). The PVE was < 4% for all 5 proteins, well below the 7.56% PVE value for HHV5 gB.

## Discussion

### Methodological considerations

Here we employed the iNeo-Epp algorithm^40^ (see “Materials and methods”) that uses a combination of diverse factors to evaluate the T-cell immunogenicity of a peptide (epitope), namely T-cell epitope prediction of the peptide-HLA Class I allele complex, factors including foreignness, accessibility, molecular weight, molecular structure, molecular conformation, chemical properties, and physical properties of target peptides. As a result, the outcome (for specified epitope/HLA Class I pairs T-cell receptor prediction, i.e. immunogenicity) includes both estimated binding affinities and immunogenicity scores; remarkably, high binding affinity is not necessarily associated with high immunogenicity, such that, e.g. an epitope with high binding affinity to a HLA Class I allele (indicated by the percentile rank value) may have a low immunogenicity (indicated by a low immunogenicity score). This is in contrast to other epitope prediction algorithms (e.g. ref^41^) which focus on the epitope-HLA allele binding affinity, which is a necessary but not sufficient step for immunogenicity.

### Brain cancer and HLA

Previous findings regarding the influence of specific viruses on brain cancer have been somewhat inconclusive. Here, we evaluated the association of 11 viruses and the population risk of brain cancer with respect to Class I HLA, a system that is critically involved in the human immune response to viruses and other foreign antigens. Specifically, we evaluated correspondence between HLA-viral antigen immunogenicity and the brain cancer HLA protection/susceptibility profile based on the premise that variability in HLA binding and immunogenicity with viral antigens influences cancer risk via differential ability to eliminate potentially oncogenic viruses. The findings demonstrated a negative association between brain cancer-HLA protection/susceptibility profile and all 11 HLA-viral antigen immunogenicities, with substantial effects (> 4% PVE) by 6 viruses (JCV, HHV6A, HHV5/CMV, HHV3/VZV, HHV8, HHV7) and negligible effects (< 1% PVE) by 5 viruses (HPV, HHV6B, HHV4, HHV2). This suggest that Class I HLA alleles that bind with, and eliminate, the former 6 viruses in particular may protect against brain cancer risk; conversely, in the absence of antigen elimination due to weak binding and/or low immunogenicity, these viruses may be involved in brain cancer risk. Since both BC-HLA and immunogenicity scores refer to the same HLA Class I alleles, and since the potential presence (and associated effects) of the various viruses are not mutually exclusive, the PVE contributions of the 6 viruses above can be added and their sum would be an estimate of how much of the variance in BC-HLA scores could be accounted for by PVE contributions of these viruses. This sum amounts to 44.5%, which leaves 55.5% of the variance of BC-HLA scores to be accounted for. It is reasonable to suppose that this would be the contribution of HLA Class II alleles, involved in the formation of antibodies against those viruses. This hypothesis remains to be investigated. Finally, it should be noted that only negligible contributions were found by HHV1, HHV2, HHV4, HHV6B, and HPV.

All four viruses identified here as most relevant to brain cancer risk, reflecting variation of their immunogenicity with common Class I HLA alleles, share several features as follows: (1) they are practically ubiquitous; (2) initial infection typically occurs during childhood; (3) following initial infection, the viruses establish lifelong latency in the host; (4) the latent viruses can be reactivated by various triggers and/or waning immunity; and (5) they are all neurotropic. Moreover, the presence of JCV, HHV5/CMV, and HHV6 DNA and/or proteins have been fairly commonly detected in brain tumors^42^. Although not indicative of causality in and of itself, the co-localization of viral components and malignancy provides compelling evidence supporting an association. The case for VZV/HHV3, however, is not quite as clear. The majority of studies investigating VZV/HHV3 in relation to brain cancer have been serological studies in which the presence of VZV antibodies and self-reported history of varicella (i.e., chicken pox) have been associated with decreased risk of brain cancer^43–45^. Research evaluating the presence of VZV/HHV3 viral proteins in brain tumors is scant^42^; therefore, additional research is warranted regarding VZV/HHV3 viral presence in relation to brain cancer. Finally, with respect to CMV/HHV5, it is worth noting that the gB glycoprotein we used (see “Materials and methods”) is “an essential glycoprotein for both in vivo and in vitro viral replication…. Furthermore, without exception, serological responses to gB can be detected in patients infected with HCMV.”^46^ In addition to gB glycoprotein, we also evaluated 5 abundant CMV proteins, including three strains of pp65, gM and gN (Table 7), all of which were negatively associated with the BC HLA immunogenetic profile but at lower strength than gB (Table 3). In fact, the combination of gB and pp65 in CMV vaccines has been found to possess an additive protective effect^47^.

### Mechanisms of virus-induced carcinogenesis

With mounting research supporting a role of viruses in cancer, several viral oncogenic mechanisms have been identified^48^. These include direct mechanisms such as those affecting genomic instability, rate of cell proliferation, resistance to apoptosis and alteration in DNA repair mechanisms as well as indirect mechanisms including immunosuppression, chronic inflammation, and chronic antigenic stimulation. The mechanisms of carcinogenesis are fairly well characterized for JCV and CMV/HHV5 given the wealth of research on their role in oncogenesis and oncomodulation. CMV appears to indirectly promote oncogenesies via chronic inflammation, interference with signaling pathways, and influencing cell motility and proliferation factors whereas JCV directly promotes oncogenesis via expression of viral T-antigen which inhibits tumor suppression, prevention of DNA repair, and chromosomal alterations^2, 8, 49^. Considerably less research has evaluated the oncogenic mechanisms of HHV6 and HHV3/VZV. Several potential oncogenic and oncomodulataroy mechanisms have been proposed or preliminarily supported for HHV6 including disruption of apoptosis, cellular transformation, immunomodulatory mechanisms, and transactivation of other viruses^8, 50^. A role of indirect oncogenic mechanisms including immune evasion and immunosuppression have been implicated for VZV^2^. Particularly for HHV6 and HHV3/VZV, additional studies are warranted to elucidate the mechanisms that may permit or promote brain cancer.

Of the 11 viruses studied, the immunogenicity of 4 of the herpes viruses and HPV were unrelated to the brain cancer-HLA protection/susceptibility profile. Interestingly HHV6A immunogenicity was clearly associated with the brain cancer-HLA protection/susceptibility profile but HHV6B was not. Despite high molecular homology, pathomechanisms (including carcinogenic mechanisms) of HHV6A and HHV6B differ^50^. The absence of findings for HHV6b and other herpes viruses studied here or HPV do not suggest that they are not implicated in brain cancer. Indeed, HPV has been shown to be associated with brain tumors^3^ and some evidence has linked HSV-1/HHV1, HHSV-2, and EBV with brain cancer^1, 4, 8^. Rather, the current findings suggest that their potential roles in brain cancer are likely unrelated to immunogenicity with common Class I HLA alleles. Whether that is potentially due to viral immune escape mechanisms including downregulation of Class I HLA molecules^2^ or the influence of other mechanisms remains to be seen.

Here we focused on Class I HLA due to its early immune response to viral infection. However, Class II HLA, which is involved in long-term immunity including antibody production and immunological memory, may also be associated with brain cancer risk and protection. HLA Class I (HLA-A, B, C) and HLA Class II (HLA-DR, DQ, DP) are complementary classes of cell surface proteins that are instrumental in binding and elimination of intracellular and extracellular foreign antigens (e.g., viruses, bacteria, cancer neoantigens), respectively. Widespread identification of CMV DNA in the peripheral blood of patients with CMV DNA-positive brain tumors has been suggested to reflect either systemic reactivation or viral shedding from tumor cells to the periphery^51, 52^ in which case Class II HLA may be particularly relevant.

In summary, across the 69 Class I HLA alleles investigated here, the immunogenicity of all 11 viruses studied were negatively associated with the BC-HLA profile such that HLA allele-epitope complexes with high immunogenicity were associated with protective (i.e., negative) population brain cancer-HLA risk scores whereas those with low immunogenicity were associated with susceptibility (i.e., positive) population brain cancer risk, accounting for 44.52% of BC-HLA variance explained. Of the 11 viruses tested, 6 (JCV, HHV6A, HHV5, HHV3, HHV8, HHV7) had a substantial effect on BC-HLA scores, contributing 95% to the BC-HLA variance explained above, whereas the remainder 5 viruses (HPV, HHV6B, HHV1, HHV4, HHV2) contributed only 5%. Although the association between brain cancer and JCV, HHV6A, HHV5 and HHV3 has been more investigated, the presence of genomic material from HHV7^53, 54^ and HHV8^55^ has been reported in the brain. A major issue has to do with the large variety of brain tumors and the reasonable possibility that different viruses might be involved in different tumors, thus making the generalization of virus(es)-BC association difficult.

Our findings speak to the interaction of viral exposure and host immunogenetics on brain cancer and may partially account for some of the conflicting findings linking these particular viruses to brain cancer risk. However, these novel finding must be considered within the context of study limitations. First, it is unclear if the current findings extend to other geographic locations outside of Continental Western Europe. Evolutionary pressure aimed at maximizing geographically-relevant pathogen elimination has shaped the distribution of HLA alleles at the population level^10, 11^. Thus, the brain cancer-HLA profile used for analyses here may differ in other countries. Second, it is uncertain to what extent these population-based findings apply to individuals. The HLA region is the most highly polymorphic region of the human genome^12^. Each individual possesses 12 classical HLA alleles (two of each gene) that determine the repertoire of foreign antigens that can be bound and subsequently eliminated. Here our analyses focused on the association between the frequencies of single HLA alleles and the population prevalence of brain cancer; the combined effects of the 12 alleles carried by an individual with regard to viral immunogenicity and brain cancer occurrence remain to be investigated. Third, the brain cancer-HLA profile was determined here for overall brain cancer risk and may vary by brain cancer subtypes. Finally, we evaluated immunogenicity with respect to representative proteins of the 11 viruses and acknowledge that other proteins may differentially relate to the brain cancer-HLA profile. Despite these limitations, HLA binding and elimination of viral antigens that have been linked to brain cancer is an interesting avenue for continued investigation, particularly in light of ongoing research on viruses as candidates for oncolytic virotherapy^4, 56–58^.

### Clinical relevance

The main focus of this study is on HLA, the common denominator for the epidemiologically derived BC HLA risk scores and the in silico derived immunogenicity scores for 11 viruses that have been implicated in nonmetastatic brain cancer. The main function of HLA is the elimination of foreign antigens, by killing the infected cell (HLA Class I, CD8+ mediated and assisted by Class II CD4+ assisted) and/or neutralizing the offending antigen by specific antibodies against it (HLA Class II, CD4+ mediated). The key and necessary step in this process is the formation of a complex between the HLA molecule and a peptide fragment (epitope) of the foreign antigen: it is this complex that is transported to the cell surface and engages the CD8+ and CD4+ lymphocytes. Therefore, the successful formation of a HLA molecule–antigen complex, and, hence, the successful lymphocyte engagement depends critically on the availability of HLA molecules which would bind to the foreign antigen. Indeed, the outcome of immune checkpoint blockade (ICB) therapy in melanoma depends on the HLA makeup of the patient^59^, in keeping with epidemiological evidence that higher population frequencies of HLA alleles found beneficial in ICB therapy are also associated with lower melanoma prevalence in those populations^27^. The results of this study form the basis of predictions with respect to outcomes of interventions against diseases caused by a virus among those we investigated (e.g. vaccines against HHV5/CMV^48^) or tumors where viral products have been found (e.g. glioblastoma^60^). It should be noted that the crucial role of HLA extends to any immunotherapy directed against tumor specific antigens (TSA; neoantigens)^61, 62^. Given this fundamental, permissive role of HLA in enabling cancer immunotherapies, it would be ideal if the requisite HLA molecule for a particular tumor TSA or virus could be present. Indeed, we proposed recently such a procedure, namely the administration of mRNA of specific HLA Class I molecules with high binding affinities to specific TSA(s)^63^. Such an approach may be particularly useful when coupled with immune checkpoint blockade for glioblastoma^64^.

## Materials and methods

### Prevalence of brain cancer

The population prevalences of brain cancer (BC) were computed for 14 countries in Continental Western Europe (Table 4). For each country we identified the total number of people with BC in 2019 from the Global Health Data Exchange^65^, a publicly available catalog of data from the Global Burden of Disease study, and divided those values by the total population of each country in 2019^65^. Life expectancy was not included in the current analyses since it is virtually identical for these countries^17^.

**Table 4:** Prevalence of brain cancer in 14 CWE countries.

	Country	Brain cancer prevalence (%)
1	Austria	0.0273
2	Belgium	0.0626
3	Denmark	0.1515
4	Finland	0.1102
5	France	0.0609
6	Germany	0.0155
7	Greece	0.0918
8	Italy	0.0313
9	Netherlands	0.0716
10	Norway	0.1319
11	Portugal	0.0261
12	Spain	0.0448
13	Sweden	0.0891
14	Switzerland	0.0614

### HLA alleles

We obtained the population frequency in 2019 of 69 common HLA Class I alleles from 14 Continental Western European Countries (Austria, Belgium, Denmark, Finland, France, Germany, Greece, Italy, Netherlands, Portugal, Norway, Spain, Sweden, and Switzerland)^66^. There was a total of 2746 entries of alleles from these countries, comprising 844 distinct alleles. Of those, 127 alleles (69 Class I and 58 Class II) occurred in 9 or more countries, with a minimum frequency (in any country) of 0.01. In this study we focused on the 69 HLA Class I alleles whose mean frequencies (over the contributing countries) are given in Table 5.

**Table 5:** The 69 HLA Class I alleles used and their mean frequencies.

Class I								
Gene A			Gene B			Gene C		
	Allele	Frequency		Allele	Frequency		Allele	Frequency
1	A*01:01	0.1170	1	B*07:02	0.1009	1	C*01:02	0.0370
2	A*02:01	0.2715	2	B*08:01	0.0791	2	C*03:03	0.0506
3	A*02:05	0.0122	3	B*13:02	0.0238	3	C*04:01	0.1349
4	A*03:01	0.1501	4	B*14:01	0.0091	4	C*05:01	0.0716
5	A*11:01	0.0527	5	B*14:02	0.0275	5	C*06:02	0.0829
6	A*23:01	0.0237	6	B*15:01	0.0544	6	C*07:01	0.1472
7	A*24:02	0.1051	7	B*15:17	0.0104	7	C*07:02	0.1020
8	A*25:01	0.0139	8	B*15:18	0.0043	8	C*07:04	0.0146
9	A*26:01	0.0356	9	B*18:01	0.0609	9	C*12:02	0.0160
10	A*29:01	0.0058	10	B*27:02	0.0070	10	C*12:03	0.0678
11	A*29:02	0.0315	11	B*27:05	0.0435	11	C*14:02	0.0231
12	A*30:01	0.0165	12	B*35:01	0.0690	12	C*15:02	0.0370
13	A*30:02	0.0132	13	B*35:02	0.0187	13	C*16:01	0.0303
14	A*31:01	0.0295	14	B*35:03	0.0261			
15	A*32:01	0.0368	15	B*35:08	0.0111			
16	A*33:01	0.0116	16	B*37:01	0.0136			
17	A*33:03	0.0066	17	B*38:01	0.0276			
18	A*36:01	0.0063	18	B*39:01	0.0146			
19	A*68:01	0.0353	19	B*39:06	0.0069			
20	A*68:02	0.0220	20	B*40:01	0.0474			
			21	B*40:02	0.0212			
			22	B*41:01	0.0087			
			23	B*41:02	0.0056			
			24	B*44:02	0.0623			
			25	B*44:03	0.0431			
			26	B*44:05	0.0054			
			27	B*45:01	0.0090			
			28	B*47:01	0.0043			
			29	B*49:01	0.0220			
			30	B*50:01	0.0164			
			31	B*51:01	0.0640			
			32	B*52:01	0.0158			
			33	B*55:01	0.0129			
			34	B*56:01	0.0075			
			35	B*57:01	0.0278			
			36	B*58:01	0.0141			

### BC-HLA protection/susceptibility (PS) scores

We estimated the association between brain prevalence and allele frequency by computing the covariance between the prevalence of brain cancer and the population frequency of the 69 HLA Class I alleles of Table 5. The covariance can be negative or positive, indicating a protective or susceptibility (PS) association, respectively. We call these covariance values PScov scores. Thus the HLA-BC profile is a vector of 69 PScov scores. The equation for the PScov scores is:

1 $$\mathrm{PScov }=\frac{1}{N-1}\sum_{i}^{i=1,N}({f}_{i}-\overline{f })({p}_{i}-\overline{p })$$

where ${f}_{i}, {p}_{i}$ denote allele frequency and BC prevalence for the *i*th country, respectively, and

$\overline{f },\overline{p }$ are their means. As mentioned above, cancer prevalence was available for each one of the 14 CWE countries but availability of allele frequencies varied across countries. Hence, we used only the 69 alleles (Table 5) whose frequencies were available in at least 9 countries.

### Random permutations test for assessing the statistical significance of the BC-HLA PScov profile

In this analysis, we tested the null hypothesis that the BC-HLA PScov profiles (vectors) may be due to chance by performing the permutation test below. Let $H$ be the observed Disease-HLA profile and $H^{\prime}$ be the profile obtained by random permutation, as follows. (1) For each allele, its observed frequencies were paired to randomly permuted prevalences of BC in the 14 CWE countries above. (2) The covariance between allele frequency and corresponding (to the permuted country) disease prevalence was computed to yield a “permuted” Disease-HLA profile, $H^{\prime}$, consisting of 69 PScov scores. (3) The observed (*H*) and permuted (*H’*) PScov profiles were ranked and the absolute differences between the *H* and *H’* ranks computed and summed: a sum of zero would indicate that the two profiles are the same. (4) Finally, we carried out this procedure 1,000,000 times and counted the number of times *M* that that sum was equal to zero, indicating that the ranks of the observed and permuted PScov scores are the same. (5) Then, the ratio $w=\frac{M}{\mathrm{1,000,000}}$ is the probability that the observed profile $H$ could be due to chance.

### Virus antigens

We estimated the immunogenicity (for each one of the 69 Class I alleles) of proteins of 11 viruses that have been implicated in brain cancer, namely 9 human herpes virus species (HHV1-HHV9), human polyoma JC virus (JCV), and human papilloma virus (HPV). Details of the proteins analyzed are given in Table 6 and their amino acid (AA) sequences are given in Appendix 1. Given the extensive research on the role HHV5/CMV in glioblastoma and the use of its protein pp65 in vaccines against glioblastoma^60^, we also estimated the HLA-related immunogenicity of the following 5 HHV5/CMV proteins (Table 7): pp65 (strain AD169), pp65 (strain Merlin), pp65 (strain Towne), gM_HCMVA (strain AD169), and gN_HCMVA (strain AD169).

**Table 6:** Viral proteins used.

Virus	Protein description	UniprotKB ID	N (AA)
HHV1	Envelope glycoprotein D	Q69091	394
HHV2	Envelope glycoprotein D	P03172	393
HHV3	Envelope glycoprotein E	Q9J3M8	623
HHV4	Envelope glycoprotein B	P03188	857
HHV5	Envelope glycoprotein B (gB)	P06473	906
HHV6A	Glycoprotein Q2	P0DOE0	214
HHV6B	Glycoprotein Q1	Q9QJ11	516
HHV7	Envelope glycoprotein H	P52353	690
HHV8	Envelope glycoprotein H	F5HAK9	730
JCV	Major capsid protein VP1	P03089	354
HPV	Major capsid protein L1	Q81007	494

Abbreviations as in Table 3.

**Table 7:** The 5 additional HHV5/CMV proteins used.

Protein	Protein description	UniprotKB ID	N (AA)
pp65 (strain AD169)	65 kDa phosphoprotein	P06725	561
pp65 (strain Merlin)	65 kDa phosphoprotein	Q6SW59	561
pp65 (strain Towne)	65 kDa phosphoprotein	P18139	551
gM_HCMVA (strain AD169)	Envelope glycoprotein M	P16733	372
gN_HCMVA (strain AD169)	Envelope glycoprotein N	P16795	138

### Determination of immunogenicity of HLA class I alleles

**Figure 9:**
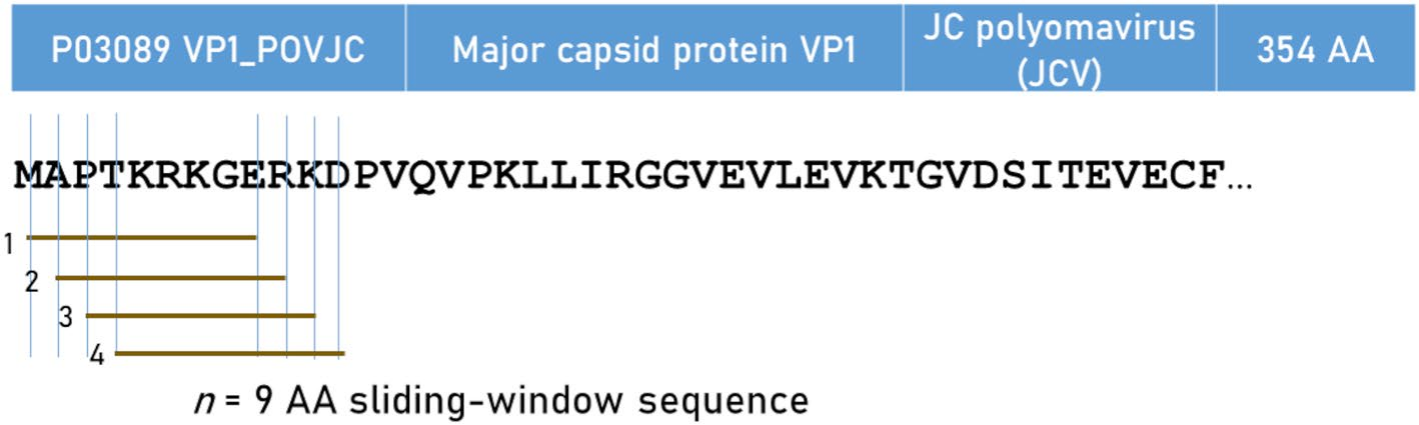
The sliding nonamer window approach used to determine exhaustively in silico the immunogenicity of all possible consecutive nonamers in a protein, illustrated here for human polyoma major capsid protein VP1.

The INeo-Epp method^40^ was used for T-cell receptor (TCR) epitope prediction using the INeo-Epp web tool via the INeo-Epp web form interface^67^. For that purpose, we split a given virus antigen (Table 6) to all possible 9-mer (nonamer) AA residue epitopes using a sliding window approach^68–70^ (Fig. 9) and submitted each epitope to the web-application together with a specific HLA allele (Table 5). More specifically, we paired all epitopes with all alleles and obtained for each pair its percentile rank, a measure of binding affinity of the epitope-HLA allele complex; smaller percentile ranks indicate higher binding affinity. The web-application gave as an outcome a TCR predictive score for pairs with high binding affinities (percentile rank < 2); scores > 0.4 indicated positive immunogenicity and were analyzed further. We computed the following as a comprehensive measure of immunogenicity for quantitative analyses. Let K be the number of nonamers that showed positive immunogenicity (score > 0.4); then, K weighted by their average score $\overline{w }$, would serve as a good estimate of the overall effectiveness of a given allele, *I,* to induce immunogenicity for a given protein:

2 $$I=\overline{w }K$$

**Figure 10:**
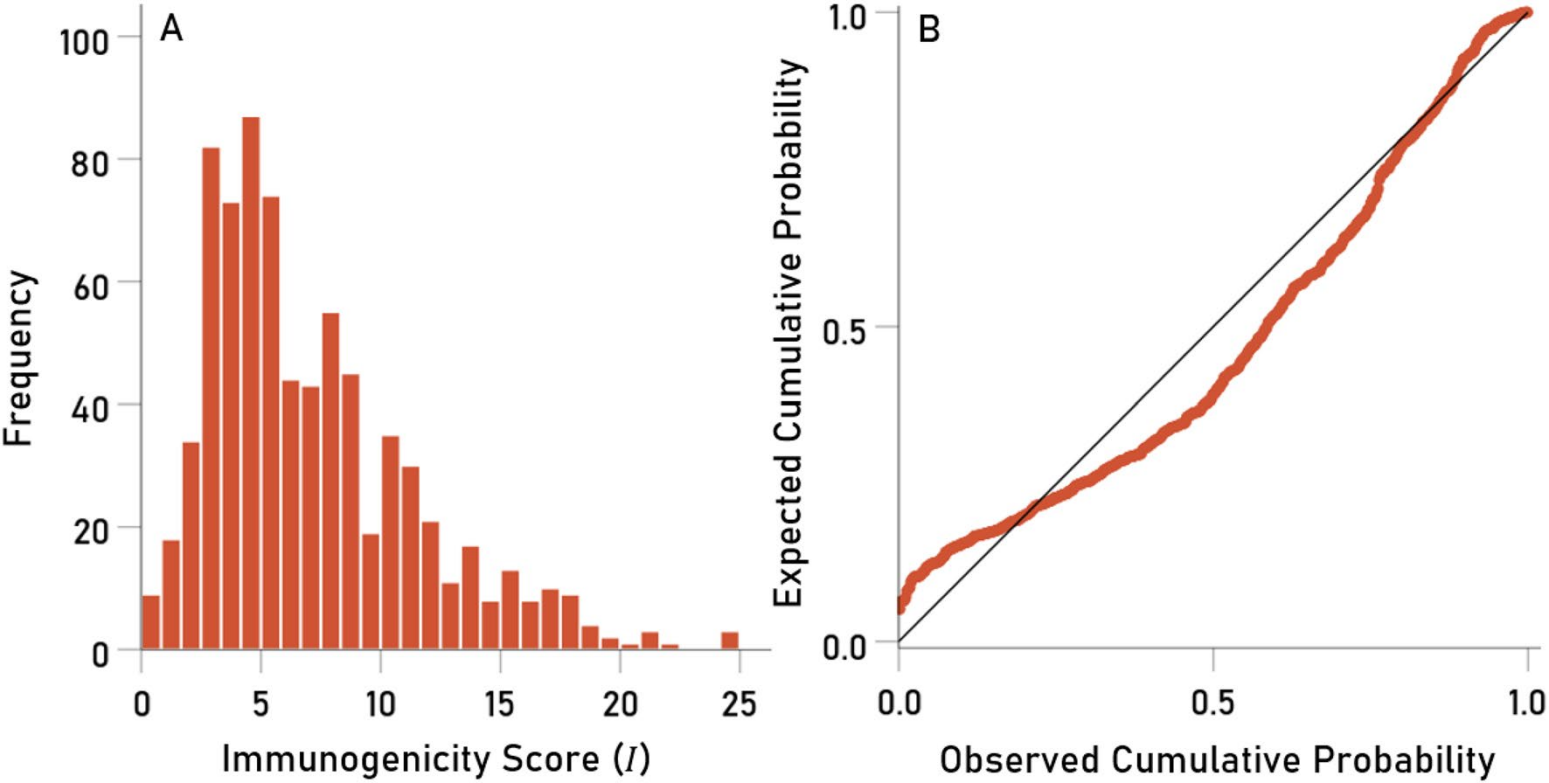
(**A**) frequency distribution of raw immunogenicity score *I* (N = 11 virus proteins × 69 HLA alleles = 759). (**B**) Probability-probability (P-P) plot to illustrate deviation from normal distribution.

**Figure 11:**
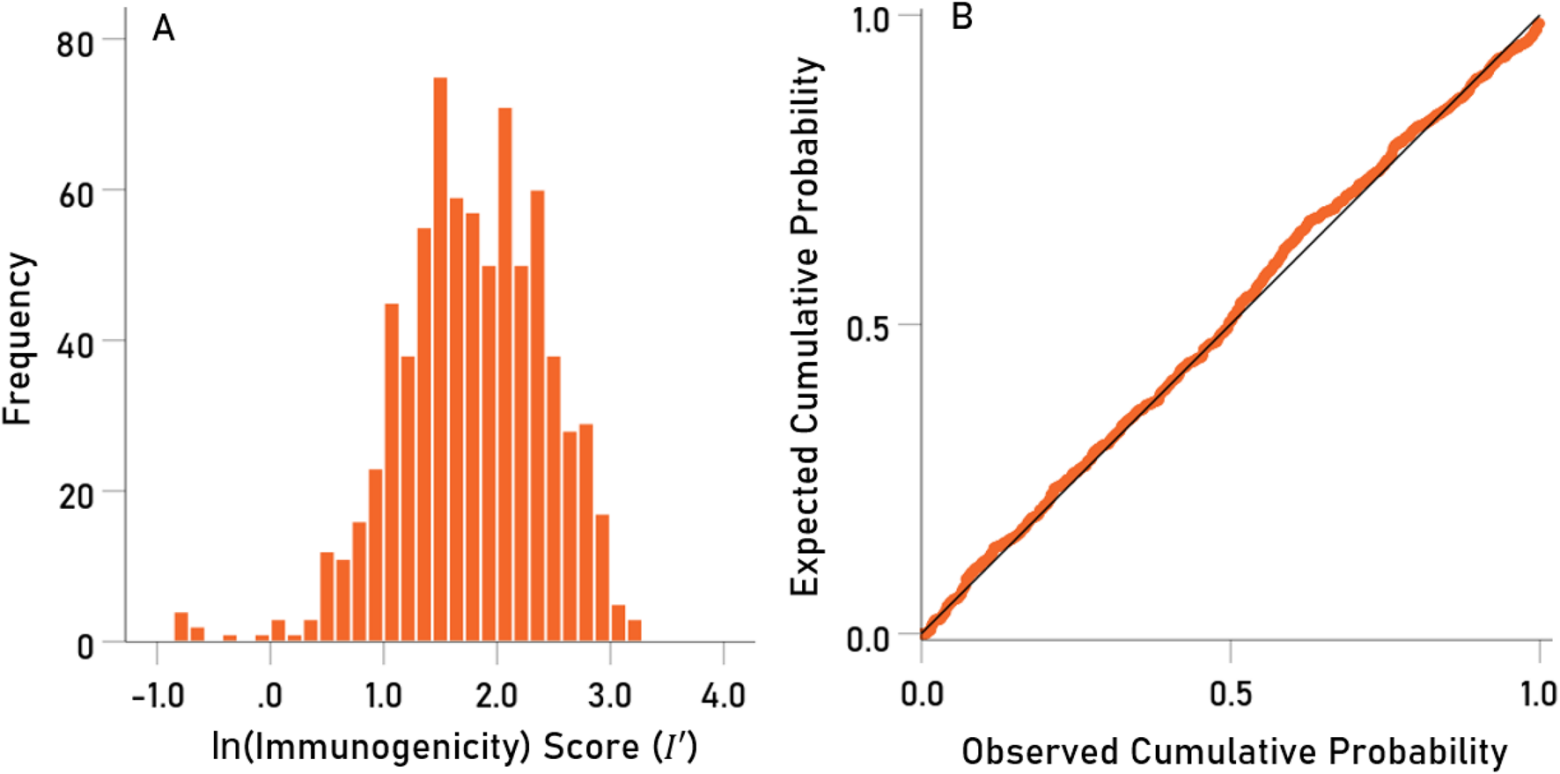
(**A**) frequency distribution of the log-transformed immunogenicity score $I^{\prime}$. (**B**) Probability-probability (P-P) plot to illustrate closeness to normal distribution.

The distribution of *I* was skewed to the right (Fig. 10A), deviating substantially from a normal distribution (Fig. 10B). A logarithmic transformation of *I* made the distribution more symmetric (Fig. 11A) and closer to normal (Fig. 11B). Therefore, this transformation was applied to *I* for further analyses:

3 $${I}^{\prime}={{\text{log}}}_{{\text{e}}}\left(I\right)={\text{ln}}(I)$$

### Association of immunogenicity with BC-HLA PScov score

We evaluated the association between BC-HLA PScov scores (Eq. 1) and virus protein immunogenicity scores $I^{\prime}$ (Eq. 3) by computing the Pearson correlation between them for each HLA allele. The correlation coefficient obtained for each virus was squared and multiplied × 100 to provide the percent of BC-HLA PScov explained (PVE) by the virus protein immunogenicity:

4 $$PVE=100{r}^{2}$$

The potential groupings of the 11 virus PVEs were explored using multidimensional scaling (MDS) and hierarchical tree clustering.

### Implementation of analysis procedures

The IBM-SPSS statistical package (version 28) was used for implementing standard statistical analyses, including descriptive statistics and regression analysis. Reported P-values are 2-sided, unless noted otherwise. For MDS, the PROXSCAL procedure was used (model: ratio, initial configuration: simplex, stress convergence: 0.0001, minimum stress: 0.0001, maximum number of iterations: 100). For hierarchical tree clustering, the average between-groups linkage was used as the method and squared Euclidean distance as the interval, implemented by the Hierarchical Cluster function. Finally, the permutations test was implemented using FORTRAN (Geany, version 1.38, built on or after 2021-10-09) and 64-bit Mersenne Twister random number generator with a large random double-precision odd-number seed.

### Supplementary Information

Supplementary Information.

## Data Availability

All data used were retrieved from freely accessible websites and, as such, are publicly and freely available [ref.^58^: http://ghdx.healthdata.org/gbd-results-tool; ref.^59^: http://allelefrequencies.net/hla6006a.asp; ref.^27^: http://www.biostatistics.online/ineo-epp/neoantigen.php].
