# Supplementary material for: Association between brain cancer immunogenetic profile and in silico immunogenicities of 11 viruses
Source: Sci Rep. 2023 Dec 6;13:21528. doi: 10.1038/s41598-023-48843-6 (PMC10700375; doi:10.1038/s41598-023-48843-6)
Supplement: Supplementary file 1 — Supplementary Information. [file 41598_2023_48843_MOESM1_ESM.docx]

**APPENDIX 1**

Amino acid sequences of the 9 HHVs, JCV, and HPV proteins tested (Table 6). Labels are from Uniprot (https://www.uniprot.org/uniprotkb/)

| HHV1: Q69091 · GD_HHV11 | Envelope glycoprotein D | 394 AA |
| --- | --- | --- |

MGGAAARLGAVILFVVIVGLHGVRSKYALVDASLKMADPNRFRGKDLPVLDQLTDPPGVRRVYHIQAGLPDPFQPPSLPITVYYAVLERACRSVLLNAPSEAPQIVRGASEDVRKQPYNLTIAWFRMGGNCAIPITVMEYTECSYNKSLGACPIRTQPRWNYYDSFSAVSEDNLGFLMHAPAFETAGTYLRLVKINDWTEITQFILEHRAKGSCKYALPLRIPPSACLSPQAYQQGVTVDSIGMLPRFIPENQRTVAVYSLKIAGWHGPKAPYTSTLLPPELSETPNATQPELAPEDPEDSALLEDPVGTVAPQIPPNWHIPSIQDAATPYHPPATPNNMGLIAGAVGGSLLAALVICGIVYWMRRHTQKAPKRIRLPHIREDDQPSSHQPLFY

| HHV2: P03172 · GD_HHV23 | Envelope glycoprotein D | 393 AA |
| --- | --- | --- |

MGRLTSGVGTAALLVVAVGLRVVCAKYALADPSLKMADPNRFRGKNLPVLDRLTDPPGVKRVYHIQPSLEDPFQPPSIPITVYYAVLERACRSVLLHAPSEAPQIVRGASDEARKHTYNLTIAWYRMGDNCAIPITVMEYTECPYNKSLGVCPIRTQPRWSYYDSFSAVSEDNLGFLMHAPAFETAGTYLRLVKINDWTEITQFILEHRARASCKYALPLRIPPAACLTSKAYQQGVTVDSIGMLPRFIPENQRTVALYSLKIAGWHGPKPPYTSTLLPPELSDTTNATQPELVPEDPEDSALLEDPAGTVSSQIPPNWHIPSIQDVAPHHAPAAPSNPGLIIGALAGSTLAVLVIGGIAFWVRRRAQMAPKRLRLPHIRDDDAPPSHQPLFY

| HHV3: Q9J3M8 · GE_VZVO | Envelope glycoprotein E | 623 AA |
| --- | --- | --- |

MGTVNKPVVGVLMGFGIITGTLRITNPVRASVLRYDDFHIDEDKLDTNSVYEPYYHSDHAESSWVNRGESSRKAYDHNSPYIWPRNDYDGFLENAHEHHGVYNQGRGIDSGERLMQPTQMSAQEDLGDDTGIHVIPTLNGDDRHKIVNVDQRQYGDVFKGDLNPKPQGQRLIEVSVEENHPFTLRAPIQRIYGVRYTETWSFLPSLTCTGDAAPAIQHICLKHTTCFQDVVVDVDCAENTKEDQLAEISYRFQGKKEADQPWIVVNTSTLFDELELDPPEIEPGVLKVLRTEKQYLGVYIWNMRGSDGTSTYATFLVTWKGDEKTRNPTPAVTPQPRGAEFHMWNYHSHVFSVGDTFSLAMHLQYKIHEAPFDLLLEWLYVPIDPTCQPMRLYSTCLYHPNAPQCLSHMNSGCTFTSPHLAQRVASTVYQNCEHADNYTAYCLGISHMEPSFGLILHDGGTTLKFVDTPESLSGLYVFVVYFNGHVEAVAYTVVSTVDHFVNAIEERGFPPTAGQPPATTKPKEITPVNPGTSPLLRYAAWTGGLAAVVLLCLVIFLICTAKRMRVKAYRVDKSPYNQSMYYAGLPVDDFEDSESTDTEEEFGNAIGGSHGGSSYTVYIDKTR

| HHV4: P03188 · GB_EBVB9 | Envelope glycoprotein B | 857 AA |
| --- | --- | --- |

MTRRRVLSVVVLLAALACRLGAQTPEQPAPPATTVQPTATRQQTSFPFRVCELSSHGDLFRFSSDIQCPSFGTRENHTEGLLMVFKDNIIPYSFKVRSYTKIVTNILIYNGWYADSVTNRHEEKFSVDSYETDQMDTIYQCYNAVKMTKDGLTRVYVDRDGVNITVNLKPTGGLANGVRRYASQTELYDAPGWLIWTYRTRTTVNCLITDMMAKSNSPFDFFVTTTGQTVEMSPFYDGKNKETFHERADSFHVRTNYKIVDYDNRGTNPQGERRAFLDKGTYTLSWKLENRTAYCPLQHWQTFDSTIATETGKSIHFVTDEGTSSFVTNTTVGIELPDAFKCIEEQVNKTMHEKYEAVQDRYTKGQEAITYFITSGGLLLAWLPLTPRSLATVKNLTELTTPTSSPPSSPSPPAPSAARGSTPAAVLRRRRRDAGNATTPVPPTAPGKSLGTLNNPATVQIQFAYDSLRRQINRMLGDLARAWCLEQKRQNMVLRELTKINPTTVMSSIYGKAVAAKRLGDVISVSQCVPVNQATVTLRKSMRVPGSETMCYSRPLVSFSFINDTKTYEGQLGTDNEIFLTKKMTEVCQATSQYYFQSGNEIHVYNDYHHFKTIELDGIATLQTFISLNTSLIENIDFASLELYSRDEQRASNVFDLEGIFREYNFQAQNIAGLRKDLDNAVSNGRNQFVDGLGELMDSLGSVGQSITNLVSTVGGLFSSLVSGFISFFKNPFGGMLILVLVAGVVILVISLTRRTRQMSQQPVQMLYPGIDELAQQHASGEGPGINPISKTELQAIMLALHEQNQEQKRAAQRAAGPSVASRALQAARDRFPGLRRRRYHDPETAAALLGEAETEF

| HHV5: P06473 · GB_HCMVA | Envelope glycoprotein B | gB | 906 AA |
| --- | --- | --- | --- |

MESRIWCLVVCVNLCIVCLGAAVSSSSTSHATSSTHNGSHTSRTTSAQTRSVYSQHVTSSEAVSHRANETIYNTTLKYGDVVGVNTTKYPYRVCSMAQGTDLIRFERNIICTSMKPINEDLDEGIMVVYKRNIVAHTFKVRVYQKVLTFRRSYAYIYTTYLLGSNTEYVAPPMWEIHHINKFAQCYSSYSRVIGGTVFVAYHRDSYENKTMQLIPDDYSNTHSTRYVTVKDQWHSRGSTWLYRETCNLNCMLTITTARSKYPYHFFATSTGDVVYISPFYNGTNRNASYFGENADKFFIFPNYTIVSDFGRPNAAPETHRLVAFLERADSVISWDIQDEKNVTCQLTFWEASERTIRSEAEDSYHFSSAKMTATFLSKKQEVNMSDSALDCVRDEAINKLQQIFNTSYNQTYEKYGNVSVFETSGGLVVFWQGIKQKSLVELERLANRSSLNITHRTRRSTSDNNTTHLSSMESVHNLVYAQLQFTYDTLRGYINRALAQIAEAWCVDQRRTLEVFKELSKINPSAILSAIYNKPIAARFMGDVLGLASCVTINQTSVKVLRDMNVKESPGRCYSRPVVIFNFANSSYVQYGQLGEDNEILLGNHRTEECQLPSLKIFIAGNSAYEYVDYLFKRMIDLSSISTVDSMIALDIDPLENTDFRVLELYSQKELRSSNVFDLEEIMREFNSYKQRVKYVEDKVVDPLPPYLKGLDDLMSGLGAAGKAVGVAIGAVGGAVASVVEGVATFLKNPFGAFTIILVAIAVVIITYLIYTRQRRLCTQPLQNLFPYLVSADGTTVTSGSTKDTSLQAPPSYEESVYNSGRKGPGPPSSDASTAAPPYTNEQAYQMLLALARLDAEQRAQQNGTDSLDGQTGTQDKGQKPNLLDRLRHRKNGYRHLKDSDEEENV

| HHV6A: P0DOE0 · GQ2_HHV6U | Envelope glycoprotein Q2 | 214 AA |
| --- | --- | --- |

MHFLVVYILIHFHAYRGMAALPLFSTLPKITSCCDSYVVINSSTSVSSLISTCLDGEILFQNEGQKFCRPLTDNRTIVYTMQDQVQKPLSVTWMDFNLVISDYGRDVINNLTKSAMLARKNGPRYLQMENGPRYLQMETRISDLFRHECYQDNYYVLDKKLQMFYPTTHSNELLFYPSEATLPSPWQEPPFSSPWPEPTFPSRWYWLLLNYTNY

| HHV6B: Q9QJ11 · GQ1_HHV6Z | Envelope glycoprotein Q1 | 516 AA |
| --- | --- | --- |

MRPPRRSAPILVCAISMATALSNATVHRDAGTVESTPPPDDEDNYTAKYYDDSIYFNIYDGTNPTPRRRTLPEIISKFSTSEMSRLGGLKAFVPVDYTPTTTLEDIEDLLNYAICDDNSCGCLIETEARXMFGDIIICVPLSAESRGVRNLKSRIMPMGLSQILSSGLGLHFSLLYGAFGSNYNSLAYMERLKPLTAMTAIAFCPMTSKLELRQNYRLEKARXNLIVNIELLKIQNHGGQTIKTLTSFAIVRKDSDGQDWETCTRFASVSIEDILRSKPAANGTCCPPRDVHHDRPTLQSSNSWTRTEYFEPWQDVVDAYVPINDNHCPNDSYVVFQTLQGHEWCSRLNKNDTKNYLSSVLAFKNALYETEELMETIGMRLASQILSLVGQRGTSIRNIDPAIVSALWHSLPEKLTTTNIKYDIASPTHMSPALXTIFIQTGTSKQRFRNAGLLMVNNIFTVQARYSKQNMFEKKIYGYEHLGQALCEGGHVFYNPRDVYFQNIKMAATEPTVVRT

| HHV7: P52353 · GH_HHV7J | Envelope glycoprotein H | 690 AA |
| --- | --- | --- |

MYFYINSLLLIVSINGWKHWNILNSSICVNEKTNQTIIQPGLITFNFHDYNETRVYQIPKCLFGYTFVSNLFDSVNFDESFDQYKHRITRFFNPSTEKAVKIYAQKFQTNIKPVSHTKTITVSFLPLFYEKDVYFANVSEIRKLYYNQYICTLSNGLTDYLFPITERCVMRHYNYLNTVFMLALTPSFFIISVETGMDDVVFIFGNVSRIFFKAPFRKSSFIYRQTVSDDLLLITKKTTIERFYPFLKIDFLDDIWKQNYDISFLIAKFNKLATVYIMEGFCGKPVNKDTFHLMFLFGLTHFLYSTRGDGLLPLLEILNTHQSIITMGRFLEKCFKMTKSHLLYPEMEKLQNFQLVDYSYITSDLTIPISAKLAFLSLADGRIVTVPQNKWKEIENNIETLYEKHKLFTNLTQPERANLFLLSEIGNSLVFQEKIKRKIHVLLASLCNPLEMYFWTHMLDNVMDIETMFSPCATATRKDLTQRVVNNILSYKNLDAYTNKVMNTLSVYRKKRLDMFKSISCVSNEQAAFLTLPNITYTISSKYILAGTSFSVTSTVISTTIIITVVPLNSTCTPTNYKYSVKNIKPIYNISSHDCVFCESLVVEYDDIDGIIQFVYIMDDKQLLKLIDPDTNFIDVNPRTHYLLFLRNGSVFEITALDLKSSQVSIMLVLLYLIIIIIVLFGIYHVFRLF

| HHV8: F5HAK9 · GH_HHV8P | Envelope glycoprotein H | 730 AA |
| --- | --- | --- |

MQGLAFLAALACWRCISLTCGATGALPTTATTITRSATQLINGRTNLSIELEFNGTSFFLNWQNLLNVITEPALTELWTSAEVAEDLRVTLKKRQSLFFPNKTVVISGDGHRYTCEVPTSSQTYNITKGFNYSALPGHLGGFGINARLVLGDIFASKWSLFARDTPEYRVFYPMNVMAVKFSISIGNNESGVALYGVVSEDFVVVTLHNRSKEANETASHLLFGLPDSLPSLKGHATYDELTFARNAKYALVAILPKDSYQTLLTENYTRIFLNMTESTPLEFTRTIQTRIVSIEARRACAAQEAAPDIFLVLFQMLVAHFLVARGIAEHRFVEVDCVCRQYAELYFLRRISRLCMPTFTTVGYNHTTLGAVAATQIARVSATKLASLPRSSQETVLAMVQLGARDGAVPSSILEGIAMVVEHMYTAYTYVYTLGDTERKLMLDIHTVLTDSCPPKDSGVSEKLLRTYLMFTSMCTNIELGEMIARFSKPDSLNIYRAFSPCFLGLRYDLHPAKLRAEAPQSSALTRTAVARGTSGFAELLHALHLDSLNLIPAINCSKITADKIIATVPLPHVTYIISSEALSNAVVYEVSEIFLKSAMFISAIKPDCSGFNFSQIDRHIPIVYNISTPRRGCPLCDSVIMSYDESDGLQSLMYVTNERVQTNLFLDKSPFFDNNNLHIHYLWLRDNGTVVEIRGMYRRRAASALFLILSFIGFSGVIYFLYRLFSILY

| Human JC polyomavirus (JCV)  P03089 VP1_POVJC | Major capsid protein VP1 | 354 AA |
| --- | --- | --- |

MAPTKRKGERKDPVQVPKLLIRGGVEVLEVKTGVDSITEVECFLTPEMGDPDEHLRGFSKSISISDTFESDSPNRDMLPCYSVARIPLPNLNEDLTCGNILMWEAVTLKTEVIGVTSLMNVHSNGQATHDNGAGKPVQGTSFHFFSVGGEALELQGVLFNYRTKYPDGTIFPKNATVQSQVMNTEHKAYLDKNKAYPVECWVPDPTRNENTRYFGTLTGGENVPPVLHITNTATTVLLDEFGVGPLCKGDNLYLSAVDVCGMFTNRSGSQQWRGLSRYFKVQLRKRRVKNPYPISFLLTDLINRRTPRVDGQPMYGMDAQVEEVRVFEGTEELPGDPDMMRYVDKYGQLQTKML

| Human papillomavirus Q81007 | Major capsid protein L1 | 494 AA |
| --- | --- | --- |

TVYLPPVPVSKVVSTDEYVARTNIYYHAGTSRLLAVGHPYFPIKKPNNNKILVPKVSGLQYRVFRIHLPDPNKFGFPDTSFYNPDTQRLVWACVGVEVGRGQPLGVGISGHPLLNKLDDTENASAYAANAGVDNRECISMDYKQTQLCLIGCKPPIGEHWGKGSPCTNVAVNPGDCPPLELINTVIQDGDMVDTGFGAMDFTTLQANKSEVPLDICTSICKYPDYIKMVSEPYGDSLFFYLRREQMFVRHLFNRAGTVGENVPDDLYIKGSGSTANLASSNYFPTPSGSMVTSDAQIFNKPYWLQRAQGHNNGICWGNQLFVTVVDTTRSTNMSLCAAISTSETTYKNTNFKEYLRHGEEYDLQFIFQLCKITLTADVMTYIHSMNSTILEDWNFGLQPPPGGTLEDTYRFVTSQAIACQKHTPPAPKEDPLKKYTFWEVNLKEKFSADLDQFPLGRKFLLQAGLKAKPKFTLGKRKATPTTSSTSTTAKRKKR

**APPENDIX 2**

Amino acid sequences of the additional 5 HHV5/CMV proteins tested (Table 7). Labels are from Uniprot (https://www.uniprot.org/uniprotkb/)

| HHV5: P06725 PP65_HCMVA | 65 kDa phosphoprotein | 561 AA |
| --- | --- | --- |

MESRGRRCPEMISVLGPISGHVLKAVFSRGDTPVLPHETRLLQTGIHVRVSQPSLILVSQYTPDSTPCHRGDNQLQVQHTYFTGSEVENVSVNVHNPTGRSICPSQEPMSIYVYALPLKMLNIPSINVHHYPSAAERKHRHLPVADAVIHASGKQMWQARLTVSGLAWTRQQNQWKEPDVYYTSAFVFPTKDVALRHVVCAHELVCSMENTRATKMQVIGDQYVKVYLESFCEDVPSGKLFMHVTLGSDVEEDLTMTRNPQPFMRPHERNGFTVLCPKNMIIKPGKISHIMLDVAFTSHEHFGLLCPKSIPGLSISGNLLMNGQQIFLEVQAIRETVELRQYDPVAALFFFDIDLLLQRGPQYSEHPTFTSQYRIQGKLEYRHTWDRHDEGAAQGDDDVWTSGSDSDEELVTTERKTPRVTGGGAMAGASTSAGRKRKSASSATACTSGVMTRGRLKAESTVAPEEDTDEDSDNEIHNPAVFTWPPWQAGILARNLVPMVATVQGQNLKYQEFFWDANDIYRIFAELEGVWQPAAQPKRRRHRQDALPGPCIASTPKKHRG

| HHV5: Q6SW59 PP65_HCMVM | 65 kDa phosphoprotein | 561 AA |
| --- | --- | --- |

MESRGRRCPEMISVLGPISGHVLKAVFSRGDTPVLPHETRLLQTGIHVRVSQPSLILVSQYTPDSTPCHRGDNQLQVQHTYFTGSEVENVSVNVHNPTGRSICPSQEPMSIYVYALPLKMLNIPSINVHHYPSAAERKHRHLPVADAVIHASGKQMWQARLTVSGLAWTRQQNQWKEPDVYYTSAFVFPTKDVALRHVVCAHELVCSMENTRATKMQVIGDQYVKVYLESFCEDVPSGKLFMHVTLGSDVEEDLTMTRNPQPFMRPHERNGFTVLCPKNMIIKPGKISHIMLDVAFTSHEHFGLLCPKSIPGLSISGNLLMNGQQIFLEVQAIRETVELRQYDPVAALFFFDIDLLLQRGPQYSEHPTFTSQYRIQGKLEYRHTWDRHDEGAAQGDDDVWTSGSDSDEELVTTERKTPRVTGGGAMASASTSAGRKRKSASSATACTAGVMTRGRLKAESTVAPEEDTDEDSDNEIHNPAVFTWPPWQAGILARNLVPMVATVQGQNLKYQEFFWDANDIYRIFAELEGVWQPAAQPKRRRHRQDALPGPCIASTPKKHRG

| HHV5: P18139 PP65_HCMVT | 65 kDa phosphoprotein | 551 AA |
| --- | --- | --- |

MASVLGPISGHVLKAVFSRGDTPVLPHETRLLQTGIHVRVSQPSLILVSQYTPDSTPCHRGDNQLQVQHTYFTGSEVENVSVNVHNPTGRSICPSQEPMSIYVYALPLKMLNIPSINVHHYPSAAERKHRHLPVADAVIHASGKQMWQARLTVSGLAWTRQQNQWKEPDVYYTSAFVFPTKDVALRHVVCAHELVCSMENTRATKMQVIGDQYVKVYLESFCEDVPSGKLFMHVTLGSDVEEDLTMTRNPQPFMRPHERNGFTVLCPKNMIIKPGKISHIMLDVAFTSHEHFGLLCPKSIPGLSISGNLLMNGQQIFLEVQAIRETVELRQYDPVAALFFFDIDLLLQRGPQYSEHPTFTSQYRIQGKLEYRHTWDRHDEGAAQGDDDVWTSGSDSDEELVTTERKTPRVTGGGAMAGASTSAGRKRKSASSATACTAGVMTRGRLKAESTVAPEEDTDEDSDNEIHNPAVFTWPPWQAGILARNLVPMVATVQGQNLKYQEFFWDANDIYRIFAELEGVWQPAAQPKRRRHRQDALPGPCIASTPKKHRG

| HHV5: P16733 GM_HCMVAT | Envelope glycoprotein M | 372 AA |
| --- | --- | --- |

MAPSHVDKVNTRTWSASIVFMVLTFVNVSVHLVLSNFPHLGYPCVYYHVVDFERLNMSAYNVMHLHTPMLFLDSVQLVCYAVFMQLVFLAVTIYYLVCWIKISMRKDKGMSLNQSTRDISYMGDSLTAFLFILSMDTFQLFTLTMSFRLPSMIAFMAAVHFFCLTIFNVSMVTQYRSYKRSLFFFSRLHPKLKGTVQFRTLIVNLVEVALGFNTTVVAMALCYGFGNNFFVRTGHMVLAVFVVYAIISIIYFLLIEAVFFQYVKVQFGYHLGAFFGLCGLIYPIVQYDTFLSNEYRTGISWSFGMLFFIWAMFTTCRAVRYFRGRGSGSVKYQALATASGEEVAVLSHHDSLESRRLREEEDDDDDEDFEDA

| HHV5: P16795 GN_HCMVA | Envelope glycoprotein N | 138 AA |
| --- | --- | --- |

MEWNTLVLGLLVLSVVAESSGNNSSTSTSATTSKSSASVSTTKLTTVATTSATTTTTTTLSTTSTKLSSTTHDPNVMRRHANDDFYKAHCTSHMYELSLSSFAAWWTMLNALILMGAFCIVLRHCCFQNFTATTTKGY
